# Closing the gaps in childhood TB detection

**DOI:** 10.5588/ijtldopen.24.0494

**Published:** 2025-02-01

**Authors:** J. Creswell, C. Colvin

**Affiliations:** ^1^ Stop TB Partnership, Innovations and Grants, Geneva, Switzerland;; ^2^ Credence Management Solutions, Support Contractor to USAID Bureau for Global Health, Office of Infectious Disease, TB Division, Washington DC, USA.

**Keywords:** childhood TB, diagnostics

## Abstract

Globally, the gap between the number of children who develop TB and those who are detected and treated is narrowing, but it remains much wider than among adults. Several new diagnostic tests and approaches have been implemented recently, and more are anticipated in the development pipeline. We examine current tools and prospects for diagnostic testing and propose a framework to guide national TB programs and partners in addressing the TB treatment coverage gap among children while highlighting promising research and programmatic interventions.

It is widely acknowledged that current case-finding approaches and underlying health systems are missing hundreds of thousands of children with TB. Every year, about 1.3 million children develop TB, which is about 12% of the estimated burden among all age groups; yet the 191,000 children who die from TB every year is 15% of the total estimated deaths from the disease.^1^ Almost all (96%) of children who die from TB were not able to access diagnosis or treatment services. Overall, as many as one in five children who develop TB will die from the disease.^2^ The difficulties of TB diagnosis among children are well documented. Children often do not present with traditional symptoms,^3^ they have extrapulmonary disease more often than adults,^4^ and among children with pulmonary TB, obtaining a sputum sample for testing is challenging, especially for younger children who may need an invasive procedure such as gastric aspiration to get a specimen. Even when children produce sputum for testing, they tend to have fewer bacilli to detect.^5^ Since laboratory testing in children with TB often produces negative results, most are diagnosed clinically,^6^ and many providers are reluctant to make such diagnoses,^7^ instead referring children to specialized institutions, meaning more time and costs for their caregivers, posing another barrier to proper care.

Multiple studies have assessed sample collection methods,^8^ diagnostic tools^9–12^ and programmatic approaches^13–15^ to improve TB detection among children. Some promising results have been documented, including studies in this series.^16^ A collective movement at global and country levels has led to some encouraging improvements for children with TB over the last 10 years, with annual notifications among children under 15 increasing from under 400,000 to almost 700,000 in 2023, the highest figure recorded. Similarly, children represented 8.5% of all people with TB notified in 2023, the first time that threshold was crossed.^17^ While these trends are favorable, the improvements in case detection are not evenly distributed across high-burden settings or children aged 0–15 years, and much remains to be done. The most significant gap in case detection is among children aged 0–4 years, who are at high risk of morbidity and mortality due to TB.^1^

Recent WHO recommendations on childhood TB provide helpful guidance for TB programs looking to improve childhood TB detection and treatment.^18,19^ The stool specimen can be tested on the Xpert (Cepheid, Sunnyvale, CA, USA) platform with minimal processing. These same guidelines propose interim treatment decision algorithms (TDAs) and corresponding scoring systems to support TB diagnosis in the absence of microbiological evidence, which was conditionally recommended for 2 years and is currently being validated in multiple high-burden settings. These approaches will help close the gap between estimated and notified cases but are not sufficient to ensure that all children with TB have access to high-quality TB diagnostics and clinical care to start appropriate treatment.

## How to improve TB diagnosis among children

While TB notifications among children have increased in recent years, much more needs to be done to improve the programmatic, technological, innovative, and clinical aspects of TB services for children. Below, we suggest a framework for considering different interventions and how they can improve TB diagnosis and treatment for children.

Three key areas of intervention need to be implemented at scale and with high quality to close the gap in childhood TB diagnosis: more children need to be identified as having presumptive TB, more sensitive tools and effective testing approaches are needed, and improved clinical diagnosis is required. For more children to be diagnosed with TB, *at least one* of these intervention areas must be addressed (see Figure).

**Figure.**
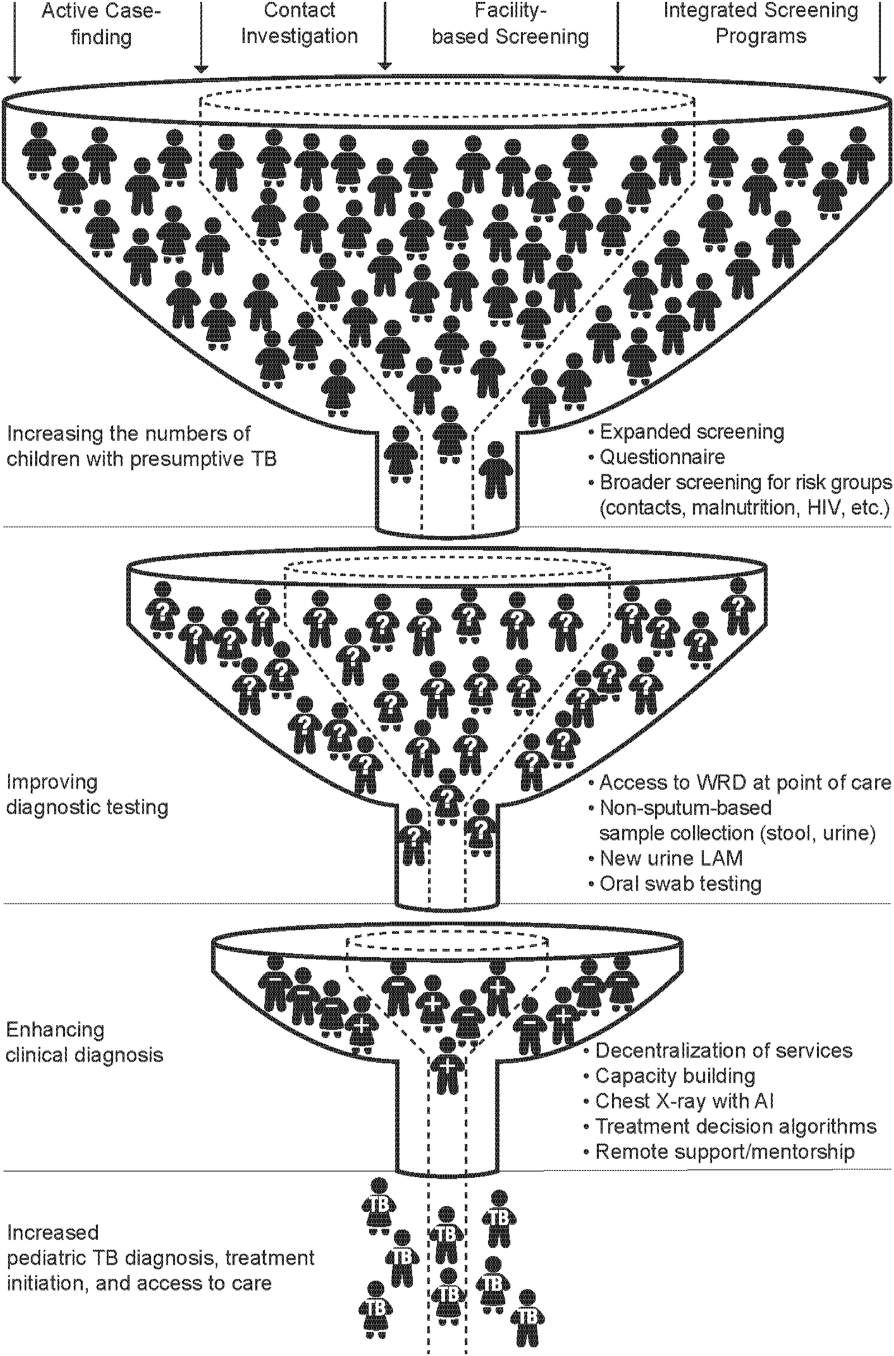
Intervention areas to improve pediatric TB detection. WRD = WHO-recommended rapid diagnostics; LAM = lipoarabinomannan; AI = artificial intelligence.

## Increasing the number of children with presumptive TB

Without large-scale efforts to identify and test more children with presumptive TB, it will be difficult, if not impossible, for national TB programs (NTPs) to detect more TB among children (while keeping factors the same, such as current laboratory testing and clinical diagnostic approaches). There are two main areas to increase the number of children identified as having presumptive TB: implementing different screening approaches and expanding the definition of presumptive TB.

Many children with TB are identified through contact investigation, which is a WHO-recommended approach and is needed.^20^ However, contact investigation alone is not enough to close the gap since many children with TB have no documented exposure and, therefore, would not be detected with this intervention.^21,22^ Moreover, despite contact investigation being a relatively low-hanging fruit, implementation of both TB detection and TB preventive treatment as critical follow-up are lagging.^1^ Systematic screening in health facilities has shown positive results^23,24^ but more research is needed on the best screening approaches.^3^ Integration of TB screening for children across disease areas and health services could also be beneficial, for example, at malnutrition centres, via maternal and child health services, and through vaccine campaigns. This will provide opportunities to screen large numbers of children for TB. NTPs will likely also need to optimize community-based efforts to screen and test children for TB, and effective approaches are likely to vary widely between and within countries.

The definition of a positive screen could be expanded to include additional symptoms that indicate presumptive TB, as well as more risk factors for TB in the country context.^25^ These include questions to determine exposure to a close source patient with confirmed TB, which is particularly important for assessing risk for drug-resistant TB (DR-TB). An expanded symptom screen would need to account for the fact that TB symptoms vary with age among children,^5^ which is likely to pose challenges for implementing screening algorithms in the various settings where they are used. Tools to identify presumptive TB, such as C-reactive protein (CRP) and chest X-ray (CXR), have been used to expand TB screening among adults and identify more people with presumptive TB, but to date, have not been shown to perform as well in screening children.^26^ For CRP, this may be due to low levels among children with primary TB.^27^ For CXR and artificial intelligence (AI), challenges stem from limited datasets to develop AI algorithms, and the difficulties in interpreting pediatric images for TB.^28^ Currently, AI to interpret CXR is recommended for people 15 years and above,^18^ and CXR has been shown to identify many asymptomatic adults who have microbiologically confirmed TB.^29,30^ CXR, with or without AI, has not been as widely used for screening children; however, as older children have similar radiographic findings as adults, expanding the current recommendations to include older children in triaging may be a possible step forward.

## Improving diagnostic testing

Once children with presumptive TB are identified, a second way to improve the number of children with a TB diagnosis would be to improve diagnostic testing. This includes multiple aspects of the testing process. Improving access to testing, specimen collection, storage, and transport, ensuring high-quality specimens of sufficient volume, and performing diagnostic tests can all increase the number of children diagnosed with TB. The difficulties in acquiring respiratory samples among children are widely understood. The use of molecular tests like Xpert^®^ MTB/RIF Ultra (Cepheid, Sunnyvale, CA, USA) or Truenat (Molbio Diagnostics, Verna, India) on a wide variety of sample types for children, including sputum (induced or not), gastric aspirates, stool, pleural fluid and urine, has been documented, but still face inherent challenges as they are not accessible for most people with TB, especially children.^1,31^ Point-of-care tests using alternate samples could make a difference. Current recommendations for urine lipoarabinomannan (LAM) testing, while expanded in 2019, are constrained to people living with HIV,^32^ and the only commercially available test that WHO has evaluated has mediocre performance. The use of urine lateral flow LAM testing could be an easier sample to collect, and some studies have shown promising results, especially among malnourished children.^33^ A newer LAM assay developed by Fujifilm (Tokyo, Japan) provided some hope for better results,^34,35^ but manufacturing concerns around the variation in lot quality^36,37^ have meant that the assay is not ready yet for field evaluations.

Several studies have looked at the potential for oral swab collection to address the shortcomings of sputum collection in children. Newer point-of-care tests using oral swab collection methods are under development and, if successful, could be highly impactful for improving TB diagnosis in children and others.^38^ However, there will likely be a loss of sensitivity in such sample collection techniques^39^ and it remains to be seen if the increased coverage of sample collection can overcome the loss in sensitivity.^40^ Cepheid’s *Mycobacterium tuberculosis* Host Response assay, which uses fingerstick whole-blood, has shown some promise in early studies and could facilitate sample collection.^41^ Even with potential advances in CXR/AI, LAM, transcriptomic signatures, and/or oral swabs, having tests that can detect drug resistance in children at high risk of exposure to DR-TB will be critical. While children with TB are missed in greater proportions than adults, the gap is even wider for DR-TB, and additional efforts are needed to start children on regimens for DR-TB.^1^

## Enhancing clinical diagnosis

Since even culture has lower sensitivity among children, any new TB diagnostic test is unlikely to achieve a level of sensitivity that allows most children to be diagnosed with microbiological confirmation. While new options for clinicians will improve laboratory testing, ultimately, it will still be critical to build competence among clinicians to diagnose TB on a clinical basis with confidence because most children will continue to be microbiologically negative. Even in studies focused on new diagnostics, most children with TB are diagnosed clinically and started on TB treatment.^42,43^ Many physicians are reluctant to make a decision to treat a child with TB (and even fewer for DR-TB) and often rely on specialists who are not readily available outside (and even within) urban centres. Decentralization of childhood TB services, including the use of clinical decision-making tools, has been shown to improve TB diagnosis and treatment initiation.^23,44^ Improving capacity and confidence for diagnosing TB in children will improve access to life-saving treatment for children and their families.

Leveraging advances in technology has the potential to improve access to better clinical diagnosis for TB. Remote support for primary care can help support decentralized management.^44,45^ The multi-country CaP-TB project built capacity among physicians and nurses to evaluate children with presumptive TB to improve clinical diagnosis. Healthcare workers reported that training, in-person coaching and ongoing supervision are critical factors for improving confidence in making clinical diagnoses. From the perspective of the person seeking care, cost barriers for follow-up visits and the cost of CXR were prominent.^46^ Similarly, TB-Speed, another multi-country project, focused on decentralization of services and improving capacity to identify children with TB through integration of screening and diagnosis with services for children with severe acute malnutrition and children living with HIV, improved management of childhood pneumonia, and use of stool samples and nasopharyngeal aspirates for diagnostic testing.^47^

CXR will continue to play a pivotal role in clinical TB diagnosis for children when microbiological tests are negative.^6^ The cost of CXR is a barrier,^48^ but even if CXR services become more accessible, there is a lack of trained readers in most high-burden countries,^49^ and there is not yet enough data to support the use of AI to address this gap in human resources. While numerous studies have documented the performance of AI software that is on par with or better than expert human readers,^50–52^ there are only a handful of published studies^28,53^ on using AI among children, and much more data is needed. A recent study showed that even a small training dataset can produce substantial improvements in performance,^28^ providing reason for optimism. However, the use case for children will likely differ, and triaging children with presumptive TB via CXR is less helpful as most will receive diagnostic tests regardless of CXR results. At the same time, expanding access to and use of CXR with or without AI to support clinical diagnosis could be highly impactful as treatment decision algorithms are so critical in children.^6^ Head-to-head assessments of AI with human readers with different levels of experience and evaluations in well-defined datasets of children with TB will be valuable.^54^

It is especially important to maintain and expand training to improve skills for clinical diagnosis as the development of diagnostic test options and guidance on multiple testing approaches^55^ may come at the expense of a careful clinical exam and use of CXR images on which many treatment decisions will still be made. The interim treatment decision algorithms (TDAs) were released by WHO in 2022^18^ and are currently under evaluation in multiple settings) and the Diagnostic CXR Atlas for Tuberculosis in Children^56^ are important resources for healthcare providers in high TB burden settings to address current gaps and ensure comprehensive evaluation of children with presumptive TB as new diagnostic tools become available.

Ideally, a combination of improved and expanded screening to identify more children for TB testing, better, more sensitive diagnostic tests that use easier-to-obtain samples, and improved and decentralized laboratory and clinical diagnosis will be needed to detect more children with TB. When developing and proposing new interventions, implementers should consider how the idea would impact each area of the framework.

## Current and future work to bring innovation to scale

There are many ongoing efforts to address the shortcomings described above; these range from introducing innovations, many that could be experimental or unproven, to bringing successful ones to scale.^44^ The Stop TB Partnership’s TB REACH initiative provides funding free from the limitations placed by other TB donors, allowing grantees to test tools and approaches that may not be WHO-recommended. Having the flexibility to evaluate new interventions and technologies helps stimulate much-needed innovation in the TB space. TB REACH-funded projects have provided data for both domestic and global policy development. Current projects include the evaluation of AI to read CXR for childhood TB and new diagnostic tests, which will provide evidence for future policy recommendations. TB REACH funding waves 10 and 11 focus on providing integrated care to people at the primary and community level.

TB REACH has consistently supported partners aiming to improve childhood TB detection, recognizing this population as critical for advancing TB diagnostic approaches. Grants targeting TB detection in children have shown great improvement in notifications among children, with TB REACH projects improving notifications by 34% during intervention periods^57^ when additional resources were deployed through various approaches. Without sustained funding, however, the gains made in childhood TB treatment and prevention will diminish when funding does as well.^58^

While expanded use of current and future testing modalities will continue to improve the numbers of children with microbiological confirmation, a greater impact will come from developing systems and a health workforce that is enabled and empowered to make clinical decisions to treat children with TB, even when laboratory tests are not available or may be negative. Improved capacity to use TDAs for clinical diagnosis and integration of TDAs in the overall diagnostic algorithm for children will be critical to this endeavour. Decentralizing childhood TB management and providing supported decision-making through remote support or video consultation can greatly improve local physicians’ ability to diagnose TB.^57^

USAID has supported multiple high-burden countries to pilot and scale up stool-based testing since the fall of 2021. These initiatives followed the interest expressed by NTPs and USAID missions after the release of the WHO guidelines on TB diagnosis, which endorsed stool testing via Xpert as a specimen for TB diagnosis.^59^ This support included a full range of activities, from the development of standard protocols and data collection forms to training on the Simple-One-Step (SOS) processing method^60^ to external supervision visits to troubleshoot implementation during the early roll-out phase. The agency also supported research to adapt the SOS method for use with the Truenat platform at the SRL in Kampala, Uganda, and conducted a pilot activity to assess the feasibility of the revised SOS protocol in routine settings in Nigeria.

Additionally, USAID provides technical assistance to NTPs to update child TB guidelines and training materials, focusing on the adaption of treatment decision algorithms to specific country contexts. Current and future research activities include a multi-country study of tongue swabs and other promising tools that are still to be decided on in the diagnostic pipeline under the Smart4TB Project. Lessons learned from these activities are used to inform the expansion of new interventions at scale, including through Global Fund grants that cover geographic areas that may not be included in USAID-funded projects.

The implementation and refinement of TDAs to strengthen the overall clinical diagnosis of children with or without access to CXR will be critical to improving both case finding and comprehensive evaluation of TB disease in children, even where microbiological confirmation is possible. Further efforts to improve clinical diagnosis include the DECIDE-TB Consortium, a multi-country effort to assess the performance of WHO and other TDAs in identifying children with TB. This project aims not only to determine which algorithms yield accurate diagnoses, but also to examine the policy and programmatic changes necessary to integrate the new practices identified.^61^

Finally, scaling up existing and novel approaches will require dedicated funding and careful planning to properly account for the additional resources needed to expand testing for children. For example, high-burden countries that scale up the use of stool-based Xpert testing need to include quantification of the cartridges needed to cover the number of tests needed to diagnose one child TB case. Ongoing surveillance and review of diagnostic algorithms and testing strategies will be needed to optimize diagnostic services, including continuous supervision to promote appropriate use of diagnostic algorithms in lower-level sites where services are decentralized. In some regions, drug susceptibility testing will need to be in place to ensure that children are on the appropriate treatment regimen and monitor treatment responses. Children and adolescents should also be fully integrated with active drug safety monitoring and management systems (aDSM) to detect adverse events and benefit from measures to address them.

## CONCLUSION

Two themes – urgency and the need for evidence – emerge in this important discussion of how to ensure effective screening and diagnosis services are available to all children with TB. On the one hand, the case detection gap and the sobering mortality data emphasize the urgency of action. At the same time, we need to ensure appropriate interpretation of the evidence for new tools and approaches in context. In the framework of identifying more children with presumptive TB, improving diagnostic testing, and enhancing clinical diagnosis, it is critical for NTPs to adapt and scale up the tools and approaches rapidly we have now, even as we wait for better options. Donors, researchers, implementers, and other key stakeholders need to ensure that children with TB are included in all efforts to improve TB detection, for example, by ensuring that children and adolescents are included in efforts to test new screening and diagnosis tools. Implementation research is needed to identify the best approaches for new diagnostics and optimizing existing tools. We must ensure that children are integrated into efforts to strengthen all programmatic efforts to improve TB detection and reach all those in need.
